# Prognostic Value of CT-Based Coronary Imaging for Perioperative Cardiovascular Risk Stratification Before Noncardiac Surgery: An Updated Systematic Review and Meta-Analysis

**DOI:** 10.3390/jcm15166290

**Published:** 2026-08-14

**Authors:** Jae Seok Bae, Jeong Yoon Jang, Yun-Ho Cho, Min Gyu Kang, Yong-Lee Kim, Hye-Ree Kim, Hyo Jin Lee, Kye-Hwan Kim, Sung-Eun Park, Jong-Hwa Ahn

**Affiliations:** 1 Division of cardiology, Department of Internal Medicine, Gyeongsang National University Changwon Hospital, School of Medicine, Gyeongsang National University, Changwon 51472, Republic of Korea; 2 Department of Internal Medicine, Gyeongsang National University Hospital, School of Medicine, Gyeongsang National University, Jinju 52727, Republic of Korea; 3 Department of Radiology, Gyeongsang National University Changwon Hospital, School of Medicine, Gyeongsang National University, Changwon 51472, Republic of Korea

**Keywords:** coronary CT angiography, coronary artery calcium, CT-derived fractional flow reserve, noncardiac surgery, perioperative cardiac risk, major adverse cardiac events

## Abstract

**Background**: Perioperative cardiovascular complications remain a major concern in patients undergoing noncardiac surgery. Coronary computed tomography (CT)-based imaging, including coronary artery calcium (CAC) scoring, coronary CT angiography (CCTA), and CT-derived fractional flow reserve (CT-FFR), enables noninvasive assessment of coronary plaque burden, anatomic stenosis, and functional ischemia. However, the comparative prognostic value of these CT-based imaging markers for predicting perioperative major adverse cardiac events (MACE) has not been systematically evaluated. **Methods**: We performed a systematic review and meta-analysis of studies evaluating CT-based coronary imaging before noncardiac surgery. PubMed, Embase, and Cochrane CENTRAL were searched from inception through December 2025. Studies were included if they assessed CAC, CCTA, or CT-FFR and reported perioperative MACE. Risk of bias was independently assessed by two reviewers using the Quality In Prognosis Studies (QUIPS) tool. Pooled effect estimates were calculated using a random-effects model. The CT-FFR analysis was pre-specified as exploratory given the limited number of eligible studies. **Results**: A total of 13 studies including 10,100 patients undergoing noncardiac surgery were included in the systematic review, and 9 studies were eligible for quantitative meta-analysis. Obstructive coronary artery disease detected by CCTA was strongly associated with perioperative MACE (pooled odds ratio [OR] 7.18, 95% confidence interval [CI] 3.89–13.25). CAC burden was also significantly associated with perioperative cardiac risk (pooled OR 2.48, 95% CI 1.76–3.50). One study evaluating CT-FFR demonstrated a strong association between CT-FFR-defined ischemia and perioperative events (OR 10.77, 95% CI 4.64–25.02). These findings suggest that different CT-based imaging markers provide complementary prognostic information, with anatomic and functional assessment offering higher point estimates than plaque burden scoring. **Conclusions**: CT-based coronary imaging markers are significantly associated with perioperative MACE in patients undergoing noncardiac surgery. CAC burden and obstructive CAD detected on CCTA demonstrated consistent prognostic associations with perioperative cardiovascular events across multiple studies. CT-FFR showed a strong exploratory signal in a single eligible study, suggesting a potential additional role for functional ischemia assessment, although further validation in larger prospective cohorts is required. CT-based coronary imaging may therefore provide valuable complementary information for perioperative cardiovascular risk stratification.

## 1. Introduction

Perioperative cardiovascular complications remain a major cause of morbidity and mortality in patients undergoing noncardiac surgery [1,2]. Current clinical guidelines recommend perioperative cardiovascular risk stratification using clinical risk indices, functional capacity assessment, and selected noninvasive testing strategies [3]. Among clinical risk models, the Revised Cardiac Risk Index remains one of the most widely used tools for perioperative risk prediction [4]. However, conventional clinical assessment alone may inadequately identify subclinical coronary artery disease and individualized ischemic risk.

Coronary computed tomography (CT)–based imaging has emerged as a comprehensive noninvasive modality for evaluation of coronary atherosclerosis and ischemia. Coronary artery calcium (CAC) scoring provides quantitative assessment of overall coronary plaque burden and has demonstrated strong prognostic value for long-term cardiovascular outcomes in multiple populations [5]. Coronary CT angiography (CCTA) enables direct visualization of coronary anatomy and obstructive coronary artery disease and has shown incremental prognostic value beyond traditional risk factors [6]. More recently, CT-derived fractional flow reserve (CT-FFR) has enabled noninvasive functional assessment of lesion-specific ischemia and demonstrated good diagnostic performance for identifying ischemia-producing coronary stenoses [7].

Several studies have investigated the prognostic value of CT-based coronary imaging in patients undergoing noncardiac surgery. Early studies demonstrated that coronary calcification is associated with increased perioperative cardiac risk [8,9]. Subsequent investigations using CCTA have shown that the presence and extent of obstructive coronary artery disease are strongly associated with perioperative cardiovascular events [10,11,12,13,14,15,16]. More recent studies using nongated chest CT demonstrated that opportunistic CAC assessment may also provide meaningful perioperative cardiovascular risk stratification [17,18]. In addition, recent evidence incorporating functional assessment with CT-FFR suggests that ischemia-related information may further improve risk stratification in this setting [19]. Our recent multicenter prospective cohort further demonstrated the incremental prognostic value of CCTA following treadmill testing in patients undergoing noncardiac surgery [20].

Despite these advances, the comparative prognostic value of different CT-based coronary imaging markers for perioperative cardiovascular risk assessment remains incompletely understood. A recent systematic review by Kyriakoulis et al. [21] focused on CCTA and CAC scoring for preoperative risk stratification, but did not include CT-FFR or incorporate the most recent prospective cohort data. In particular, the prognostic role of functional ischemia assessed by CT-FFR has not been systematically evaluated in the perioperative setting, and its incremental value over anatomic markers remains unknown.

Therefore, we performed an updated systematic review and meta-analysis to evaluate the prognostic value of CT-based coronary imaging markers—including CAC, CCTA, and CT-FFR—for predicting perioperative MACE in patients undergoing noncardiac surgery. This review extends prior work by incorporating CT-FFR data and the most recently published prospective cohort studies, providing the most comprehensive synthesis to date of CT-based coronary imaging in the perioperative setting.

## 2. Methods

### 2.1. Study Design and Reporting Standards

This study was conducted as a systematic review and meta-analysis to evaluate the prognostic value of CT-based coronary imaging markers for predicting perioperative MACE in patients undergoing noncardiac surgery. The study was performed in accordance with the PRISMA guidelines [22].

### 2.2. Literature Search Strategy

A systematic literature search was performed using PubMed, Embase, and Cochrane CENTRAL from database inception through December 2025. The search strategy combined terms related to coronary CT imaging and perioperative cardiovascular risk assessment.

The following search terms were used:

(“coronary CT angiography” OR “CCTA” OR “coronary calcium” OR “coronary artery calcium” OR “CT-FFR” OR “FFR-CT” OR “CTFFR” OR “FFRCT” OR “fractional flow reserve computed tomography”)

AND

(“noncardiac surgery” OR “non-cardiac surgery” OR “perioperative”)

AND

(“cardiac events” OR “major adverse cardiac events” OR “MACE” OR “prognosis” OR “prognostic value” OR “predictive value” OR “perioperative myocardial injury”).

In addition, the reference lists of eligible studies and relevant review articles were manually screened to identify additional potentially eligible studies.

### 2.3. Eligibility Criteria

Studies were included if they met the following criteria:

1. Patients undergoing noncardiac surgery
2. Preoperative assessment using CT-based coronary imaging, including
  ○ CAC
  ○ CCTA
  ○ CT-FFR
3. Reporting of perioperative cardiovascular outcomes, including MACE
4. Observational cohort studies or prospective clinical studies
5. Reporting sufficient data to evaluate the association between CT findings and perioperative outcomes (defined as reporting a point estimate [OR, HR, or RR] with corresponding 95% CI, or sufficient raw data to calculate such an estimate)

Studies were excluded if they:

- included cardiac surgery populations
- did not evaluate CT-based coronary imaging
- did not report perioperative outcomes
- were review articles, editorials, or case reports

Two investigators independently screened titles and abstracts, followed by full-text review to determine study eligibility. Discrepancies were resolved by consensus.

### 2.4. Data Extraction

For each eligible study, the following information was extracted:

- first author and publication year
- country and study design
- study population and type of surgery
- sample size
- CT imaging modality
- CT-derived variables (e.g., CAC, obstructive CAD on CCTA, CT-FFR)
- follow-up duration
- reported perioperative cardiovascular outcomes

These data were summarized in Table 1.

### 2.5. Risk of Bias Assessment Methodology

The risk of bias of the included studies was assessed using the QUIPS tool [23]. Risk of bias was assessed independently by two reviewers (J.Y.J. and J.S.B.); disagreements were resolved by discussion and consensus, with arbitration by a senior author (J.-H.A.) when needed. This tool evaluates six domains:

1. study participation
2. study attrition
3. prognostic factor measurement
4. outcome measurement
5. confounding adjustment
6. statistical analysis and reporting

Each domain was classified as low, moderate, or high risk of bias. The overall risk of bias for each study was determined based on the domain assessments. The results of this assessment are presented in Table 2.

### 2.6. Outcomes

The primary outcome of interest was perioperative MACE. Definitions of MACE varied slightly across studies but generally included a composite of:

- cardiac death
- myocardial infarction
- unstable angina
- urgent coronary revascularization
- or other clinically significant cardiovascular events occurring during the perioperative period.

Most studies reported outcomes within 30 days after surgery. To address heterogeneity in outcome definitions, a pre-specified outcome hierarchy was applied: 30-day outcomes were preferred over in-hospital outcomes; hard cardiac endpoints (cardiac death and myocardial infarction) were considered the primary endpoint hierarchy, with broader composite MACE definitions as secondary. When multiple outcome definitions were available from a single study, the definition closest to this hierarchy was selected for the primary analysis. The specific MACE components and the approach to preoperative revascularisation in each study are detailed in Table S1 (Supplementary Material).

### 2.7. Statistical Analysis

Separate meta-analyses were performed for each CT-based imaging marker when two or more studies evaluated the same prognostic factor.

The following associations were evaluated:

- CAC
- obstructive CAD on CCTA
- functional ischemia assessed by CT-FFR

Effect estimates were extracted as ORs or HRs with corresponding 95% CIs. Where studies reported HRs, these were included in the pooled analysis alongside ORs. This approach is considered acceptable when event rates are low (generally <10%), as the HR approximates the OR under these conditions; the event rates observed across included studies (range 2–9%) supported this approximation. Studies reporting both adjusted and unadjusted estimates were prioritized for adjusted estimates. Regarding prognostic factor definitions: for CCTA-based analyses, obstructive CAD was defined as stenosis ≥50% on visual assessment; studies using alternative thresholds (≥70%) were included in a pre-specified sensitivity analysis. For CAC analyses, studies were categorized based on the comparison reported (any CAC [score >0] vs. none, or higher vs. lower CAC categories); where studies used different thresholds (CAC ≥100, ≥400), results were synthesized using the reported study-specific dichotomization, and harmonization was addressed in sensitivity analyses restricted to studies using comparable thresholds.

Pooled effect estimates were calculated using a random-effects model (DerSimonian–Laird method) to account for potential heterogeneity across studies [24].

Between-study heterogeneity was assessed using:

- the I^2^ statistic, and
- the Cochran Q test.

Heterogeneity was considered low when I^2^ < 25%, moderate when 25–50%, and high when >50%.

Forest plots were generated to visualize the pooled associations for:

- obstructive CAD on CCTA
- CAC
- CT-FFR

When only a single study was available for a given imaging modality, pooled meta-analysis was not performed, and the study result was reported descriptively. Sensitivity analyses were planned when sufficient data were available, including: (1) restriction to studies at low overall risk of bias according to the QUIPS tool; (2) restriction to studies reporting 30-day outcomes only; (3) restriction to adjusted effect estimates; (4) restriction to studies using comparable CAC or CCTA thresholds; and (5) stratified analyses according to effect measure type (OR vs. HR). Publication bias was assessed using funnel plot asymmetry and Egger’s test when five or more studies were available for a given analysis. For analyses with fewer studies, the potential for small-study effects was acknowledged as a limitation.

### 2.8. AI Statement

During the preparation of this manuscript, the authors used Claude (Anthropic, Claude Sonnet 5) to assist in organizing and verifying bibliographic reference information. The authors have reviewed and edited the output and take full responsibility for the content of this publication.

## 3. Results

### 3.1. Study Selection

**Figure 1:**
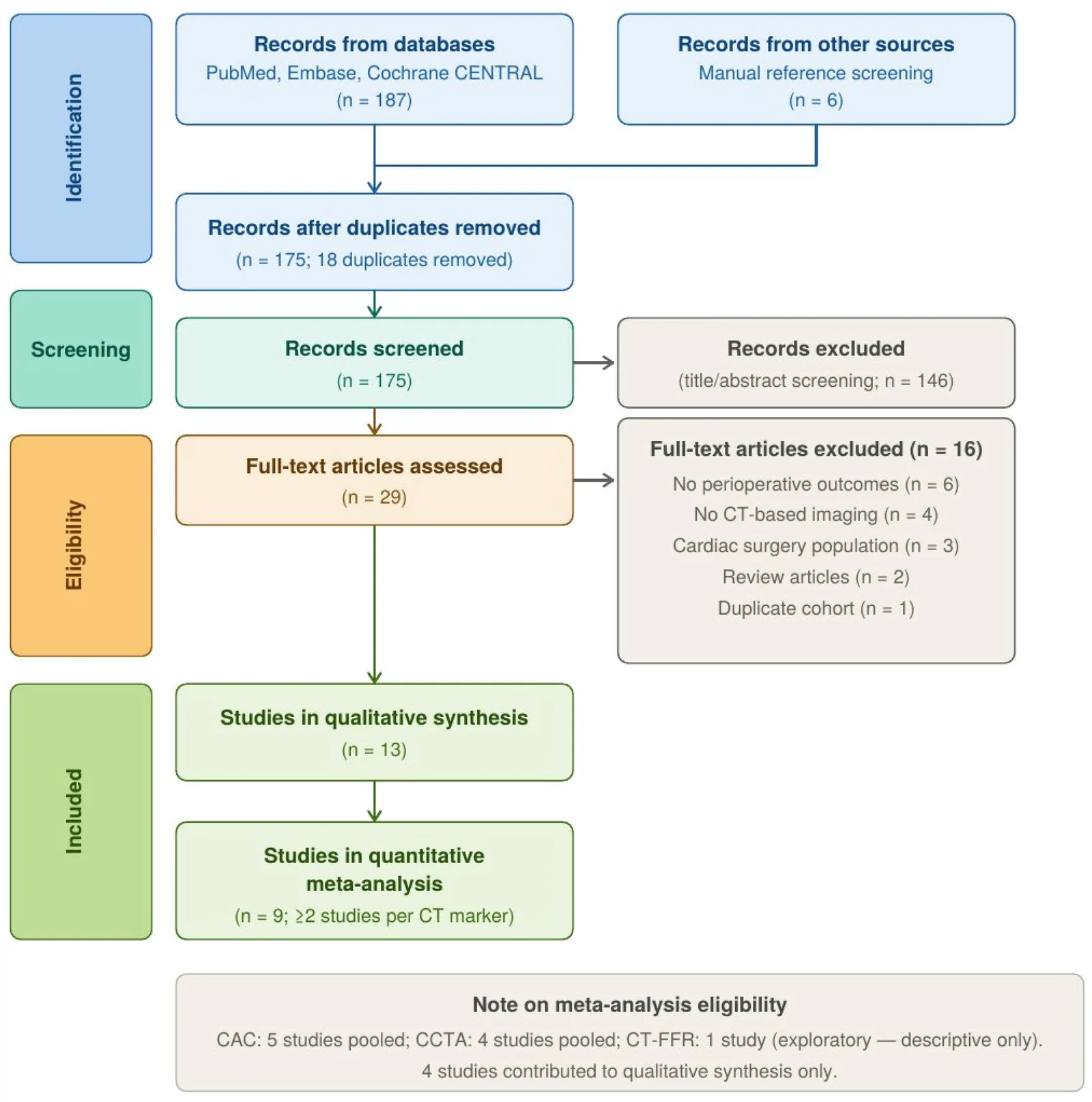
PRISMA Flow Diagram of Study Selection. Flow diagram illustrating the study selection process according to the PRISMA guidelines. A total of 187 records were identified through database searching, and 6 additional records were identified through manual reference screening. After removal of duplicates and screening of titles and abstracts, 29 full-text articles were assessed for eligibility. Ultimately, 13 studies were included in the qualitative synthesis, and 9 studies were included in quantitative meta-analysis.

The study selection process is summarized in Figure 1. A total of 187 records were identified through database searching of PubMed, Embase, and Cochrane CENTRAL, and 6 additional records were identified through manual screening of reference lists. After removal of 18 duplicate records, 175 studies remained for title and abstract screening.

Among these, 146 records were excluded after title and abstract review. The full texts of 29 articles were assessed for eligibility. Sixteen studies were excluded for the following reasons: lack of perioperative outcome data (*n* = 6), absence of CT-based coronary imaging (*n* = 4), inclusion of cardiac surgery populations (*n* = 3), review articles (*n* = 2), and duplicate cohort publication (*n* = 1).

Ultimately, 13 studies were included in the qualitative systematic review [8,9,10,11,12,13,14,15,16,17,18,19,20]. Among these, 9 studies were eligible for quantitative meta-analysis in which at least two studies evaluated the same CT-based coronary imaging marker.

### 3.2. Study Characteristics

The characteristics of the included studies are summarized in Table 1. A total of 13 studies published between 2001 and 2026 were included, comprising 10,100 patients undergoing evaluation before noncardiac surgery [8,9,10,11,12,13,14,15,16,17,18,19,20]. MACE definitions and the handling of elective preoperative revascularisation for each study are provided in Table S1.

The study populations included patients undergoing vascular surgery, liver transplantation, lung cancer surgery, diabetic lower extremity amputation, and intermediate-risk noncardiac surgery. Most studies had a prospective observational design, whereas several recent large-scale cohorts were retrospective analyses.

The CT-based coronary imaging modalities evaluated across the studies included:

- CAC assessment in six studies [8,9,11,17,18,20]
- CCTA was evaluated in seven studies [10,12,13,14,15,16,20], of which four provided sufficient data for quantitative pooling of obstructive CAD and perioperative MACE [10,12,13,20].
- CT-FFR in one recent study [19]

Most studies evaluated perioperative major adverse cardiac events within 30 days after surgery, although follow-up durations ranged from the immediate perioperative period to 40 days.

### 3.3. Risk of Bias Assessment

Risk of bias was evaluated using the QUIPS tool and is summarized in Table 2.

Most studies demonstrated low risk of bias in domains related to prognostic factor measurement and outcome measurement. Several studies showed moderate risk of bias in study participation and confounding adjustment, particularly among retrospective analyses and earlier observational cohorts.

Overall, the included studies were judged to have low to moderate risk of bias, supporting the validity of the pooled analyses.

### 3.4. Association Between Obstructive CAD on CCTA and Perioperative MACE

Among the studies evaluating CCTA, four reported sufficient data to be included in the quantitative meta-analysis assessing obstructive CAD and perioperative MACE [10,12,13,20]. The pooled analysis demonstrated that obstructive CAD detected on CCTA was strongly associated with increased perioperative risk.

**Figure 2:**
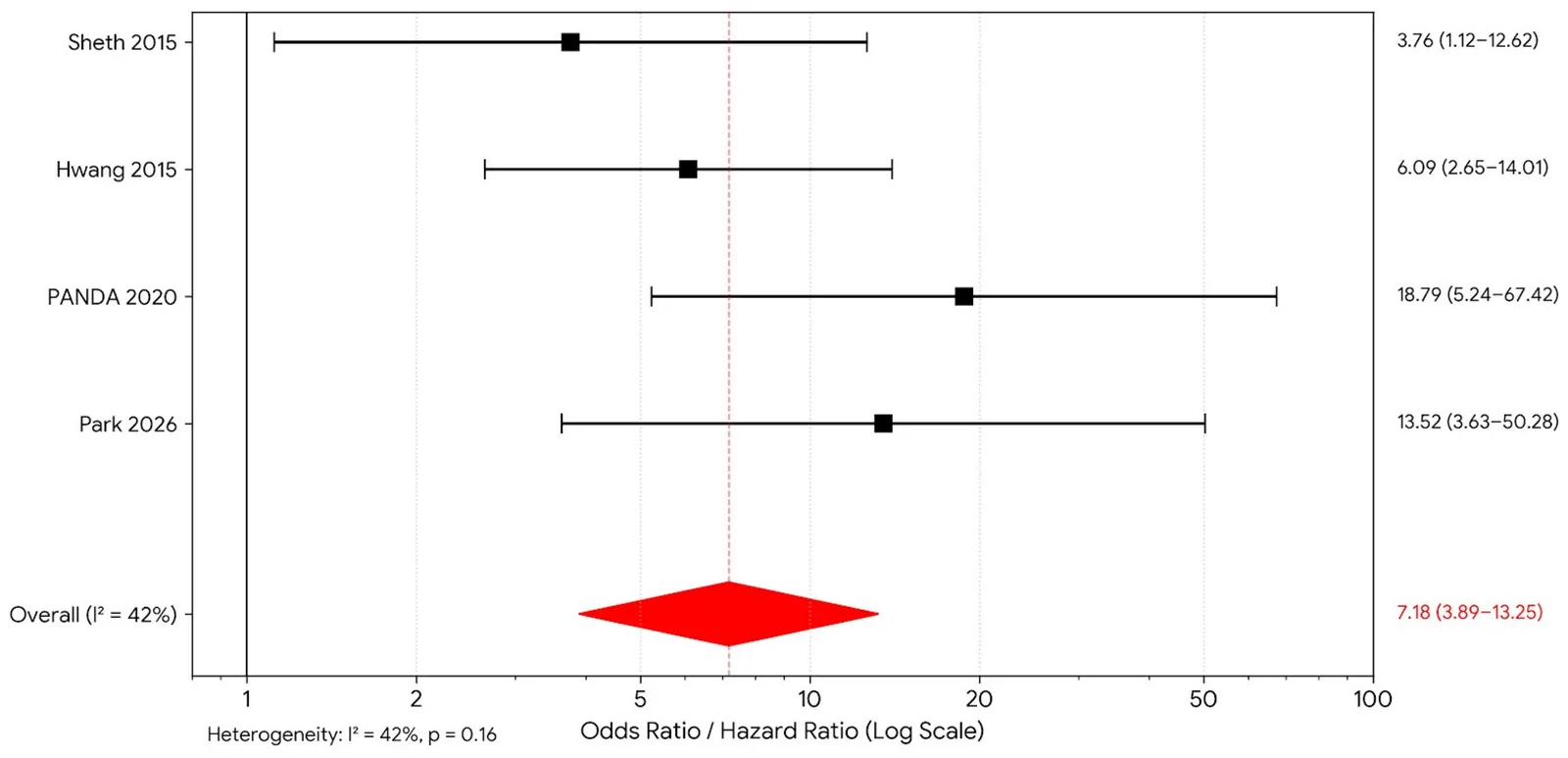
Forest Plot of Obstructive CAD on CCTA and Perioperative MACE [13,14,16,21]. Forest plot showing the association between obstructive CAD detected by CCTA and perioperative MACE. Pooled effect estimates were calculated using a random-effects model. Obstructive CAD on CCTA was significantly associated with an increased risk of perioperative events (pooled OR 7.18, 95% CI 3.89–13.25). Moderate heterogeneity was observed among studies (I^2^ = 42%). Squares represent point estimates with size proportional to study weight, horizontal lines indicate 95% confidence intervals, and the dashed vertical line represents the line of no effect (OR = 1).

The pooled effect estimate showed a significant association with perioperative MACE (OR 7.18, 95% CI 3.89–13.25) (Figure 2).

Moderate heterogeneity was observed among the studies (I^2^ = 42%, *p* = 0.16), but the direction of association was consistent across all studies.

### 3.5. Association Between CAC and Perioperative MACE

**Figure 3:**
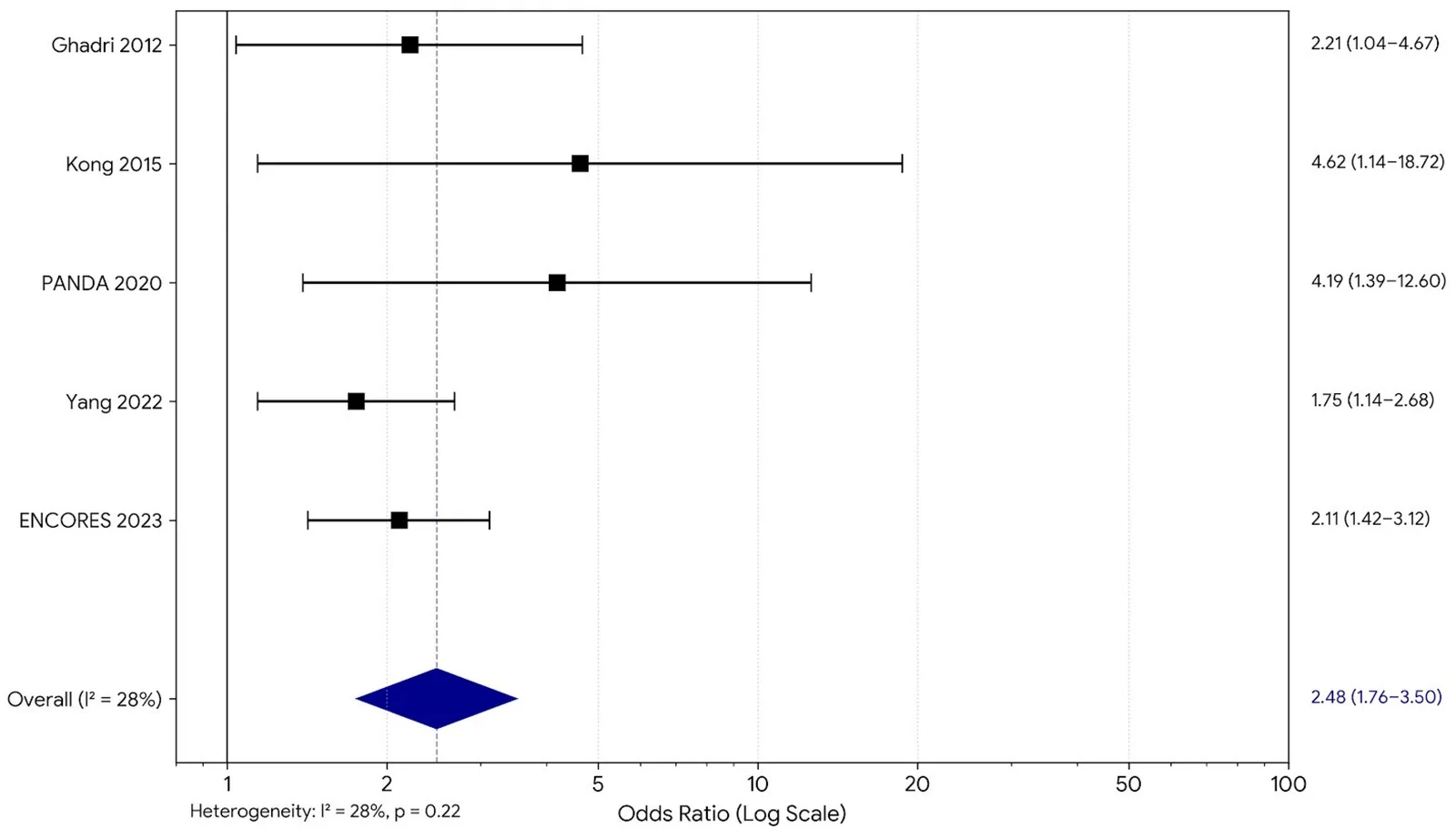
Forest Plot of CAC and Perioperative MACE [9,11,16,17,18]. Forest plot illustrating the association between CAC burden and perioperative MACE. Studies evaluating CAC derived from dedicated CAC scoring or visually estimated CAC from nongated CT were included. Increased CAC burden was significantly associated with perioperative cardiac risk (pooled OR 2.48, 95% CI 1.76–3.50). Heterogeneity among studies was low (I^2^ = 28%). Squares represent point estimates with size proportional to study weight, horizontal lines indicate 95% confidence intervals, and the dashed vertical line represents the line of no effect (OR = 1).

Five studies evaluated the association between CAC burden and perioperative MACE [8,9,11,17,18]. The pooled analysis demonstrated that increased CAC burden was significantly associated with perioperative cardiac risk, with a pooled OR of 2.48 (95% CI 1.76–3.50) (Figure 3). Between-study heterogeneity was low (I^2^ = 28%, *p* = 0.22), indicating consistent findings across studies.

### 3.6. CT-FFR and Perioperative MACE

**Figure 4:**
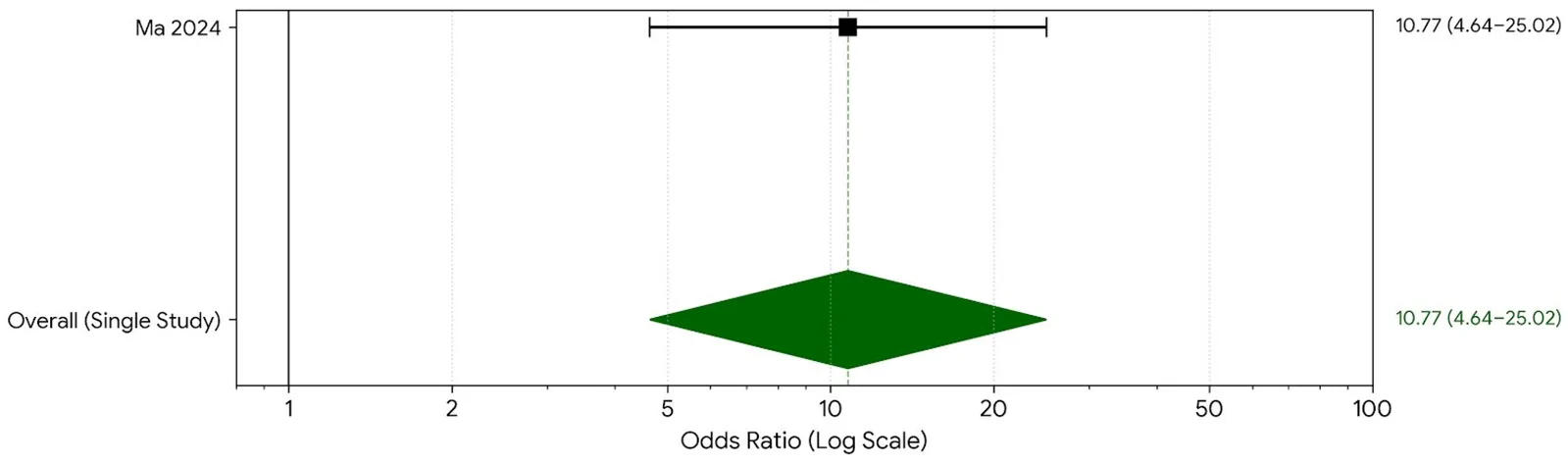
Association Between CT-FFR and Perioperative MACE (Single Study—Exploratory) [19]. Association between CT-FFR-defined ischemia and perioperative MACE. Only one eligible study evaluated CT-FFR in the perioperative setting. CT-FFR-defined ischemia demonstrated a strong association with perioperative events (OR 10.77, 95% CI 4.64–25.02). Because only a single study was available, pooled meta-analysis was not performed. Squares represent point estimates with size proportional to study weight, horizontal lines indicate 95% confidence intervals, and the dashed vertical line represents the line of no effect (OR = 1).

One study evaluated the association between CT-FFR and perioperative MACE [19]. In this study, CT-FFR-defined ischemia showed a strong association with perioperative events (OR 10.77, 95% CI 4.64–25.02) (Figure 4). Because only a single study was available, pooled meta-analysis was not performed, and these findings should be interpreted as exploratory and hypothesis-generating rather than as definitive evidence. Accordingly, this estimate cannot be directly compared with the pooled ORs derived from multiple studies for CAC and CCTA.

### 3.7. Comparative Prognostic Impact of CT-Based Imaging Markers

A comparison of pooled effect estimates across imaging modalities is shown in Table 3. The prognostic strength increased progressively across CT-based coronary imaging markers.

Specifically, CAC was associated with a pooled OR of 2.48, obstructive CAD detected on CCTA with a pooled OR of 7.18, and CT-FFR-defined ischemia with an OR of 10.77.

These findings are consistent with a pattern of increasing prognostic estimates across CT-based coronary imaging markers; however, a formal hierarchical relationship cannot be established given that the CT-FFR estimate derives from a single exploratory study and was not subject to pooled meta-analysis.

## 4. Discussion

In this systematic review and meta-analysis, we evaluated the prognostic value of CT-based coronary imaging markers for predicting perioperative MACE in patients undergoing noncardiac surgery. The principal findings of this study are threefold. First, obstructive CAD detected on CCTA was strongly associated with perioperative MACE. Second, CAC burden also demonstrated a significant association with perioperative cardiac risk, although the effect size was smaller. Third, CT-FFR-defined ischemia showed a numerically higher point estimate in a single exploratory study; however, given the limited evidence base, this finding requires confirmation before firm conclusions can be drawn.

Our findings suggest a pattern of numerically increasing effect estimates across CT-derived imaging markers, from plaque burden (CAC) to anatomic stenosis (CCTA) and, in a single exploratory study, to functional ischemia (CT-FFR). While this pattern is conceptually consistent with the underlying pathophysiological progression of coronary atherosclerosis, a formally established hierarchical relationship cannot be concluded from the available data, and the CT-FFR estimate should be viewed as hypothesis-generating.

### 4.1. Prognostic Value of CAC

CAC reflects the overall coronary plaque burden and has been widely established as a predictor of long-term cardiovascular risk in asymptomatic populations [5]. In the perioperative setting, early studies demonstrated that increased coronary calcification was associated with higher rates of perioperative myocardial injury and cardiac events [8,9]. More recent studies using both dedicated CAC scoring and visually estimated CAC from nongated CT have further supported its prognostic value before noncardiac surgery [17,18].

In the present analysis, CAC was associated with an approximately 2.5-fold increase in perioperative MACE, with relatively low heterogeneity across studies. These findings suggest that CAC provides clinically meaningful information for perioperative risk stratification, particularly as a marker of underlying coronary atherosclerotic burden. However, CAC alone does not directly assess the presence of flow-limiting coronary stenosis, which may explain the more modest effect size compared with CCTA-based assessment.

### 4.2. Prognostic Value of CCTA

CCTA enables direct visualization of coronary anatomy, allowing identification of obstructive CAD and assessment of the extent of atherosclerotic disease. Several studies have demonstrated that obstructive CAD detected on CCTA is strongly associated with perioperative cardiac events [10,11,12,13,20].

In the current meta-analysis, obstructive CAD on CCTA was associated with a more than sevenfold increase in perioperative MACE. This strong association highlights the importance of anatomic coronary disease in determining perioperative cardiovascular risk. Compared with clinical risk scores alone, CCTA provides direct information regarding coronary stenosis and plaque distribution, which may improve risk stratification in patients undergoing noncardiac surgery.

Previous studies have also suggested that CCTA may offer incremental prognostic value beyond traditional risk assessment tools and functional testing in selected patients [10,13]. In particular, detection of significant CAD may identify patients who require more intensive perioperative monitoring or optimization of medical therapy.

### 4.3. Potential Role of CT-FFR

CT-FFR represents an emerging technology that allows functional assessment of coronary lesions based on computational analysis of CCTA datasets. While CCTA identifies anatomic stenosis, CT-FFR provides information regarding the hemodynamic significance of coronary lesions, which may more directly reflect myocardial ischemia.

In the present study, CT-FFR showed the largest point estimate for predicting perioperative MACE, although evidence was limited to a single eligible study [19]. This finding should be interpreted with caution and considered exploratory rather than definitive. Direct comparison of this single-study OR with the pooled ORs derived from multiple studies for CAC and CCTA is methodologically limited, and the concept of a clear hierarchy across modalities is not firmly supported by the current data. Nonetheless, the result is conceptually consistent with the premise that functional ischemia may be a stronger predictor of adverse cardiovascular outcomes than anatomic stenosis alone, warranting dedicated prospective evaluation in larger cohorts.

Although additional studies are needed, CT-FFR may represent a promising tool for integrated anatomical and functional risk assessment in patients undergoing noncardiac surgery.

### 4.4. Clinical Implications

Based on the present findings, CT-based coronary imaging appears most appropriately positioned within a hierarchical preoperative risk assessment strategy rather than as a universal screening tool. Initial risk stratification should follow guideline-recommended steps: clinical risk scoring using the Revised Cardiac Risk Index (RCRI), assessment of functional capacity, evaluation of biomarkers such as high-sensitivity troponin or BNP/NT-proBNP, and consideration of surgical risk category [3]. CT-based coronary imaging may then be selectively applied in patients who remain at elevated or indeterminate risk after initial evaluation, particularly those with intermediate-to-high RCRI scores, limited or unassessable functional capacity, inconclusive or unfeasible stress testing, or a high pretest probability of obstructive coronary artery disease.

The detection of coronary atherosclerosis on CT imaging does not in itself mandate further diagnostic workup or coronary revascularisation. This principle, well established in stable coronary artery disease, is equally applicable in the perioperative setting: the 2022 ESC and 2024 AHA/ACC guidelines both emphasise that preoperative coronary revascularisation should not be performed solely to reduce perioperative risk unless independent indications exist [3]. For patients with abnormal CCTA findings, subsequent management should be guided by clinical context: additional noninvasive functional testing to assess inducible ischaemia may be considered in patients with intermediate anatomic stenosis where haemodynamic significance is uncertain, while invasive coronary angiography should generally be reserved for those with high-risk anatomical features (e.g., left main or proximal three-vessel disease) or specific clinical indications. Optimisation of guideline-directed medical therapy and enhanced perioperative monitoring represent the primary management response to abnormal CT findings in most patients.

Beyond risk prognostication, the overall clinical impact of CT-based coronary imaging on the perioperative work-up warrants consideration. Several included studies demonstrated that CCTA findings altered clinical management through intensified perioperative monitoring, optimisation of medical therapy, or, in selected cases, deferral or modification of the planned surgical procedure [10,12,13,20]. However, the incremental prognostic value of CT-based imaging over established clinical risk models—such as the RCRI combined with biomarkers—has not been formally evaluated in the included studies, and this represents an important gap for future research. Similarly, data on the cost-effectiveness or cost-utility of preoperative CT-based coronary imaging are currently lacking. Dedicated health-economic analyses will be required before routine incorporation of these modalities into preoperative pathways can be justified from a healthcare system perspective.

CT-based coronary imaging may be particularly valuable in specific patient subgroups at elevated perioperative cardiovascular risk. Patients with diabetes mellitus, who are prone to silent coronary artery disease and blunted ischaemic symptoms, may derive particular benefit from objective anatomic or functional coronary assessment. Patients with chronic kidney disease represent another high-risk group in whom conventional cardiac stress testing may be less reliable and in whom subclinical atherosclerosis is prevalent. Additionally, patients with limited or unassessable functional capacity—in whom the standard stress-test approach is impractical—and those with inconclusive stress-test findings or a high pretest probability of coronary artery disease are candidates in whom CT-based imaging may provide meaningful incremental information to guide perioperative decision-making. Prospective studies enriching these subgroups are needed to define the populations most likely to benefit from preoperative CT-based coronary imaging.

### 4.5. Study Limitations

Several limitations of this study should be acknowledged. First, the included studies were predominantly observational cohorts, and therefore residual confounding cannot be completely excluded. Second, the definitions of perioperative MACE varied across studies; this represents an important source of clinical heterogeneity, as differences in MI definition, inclusion of revascularization, and outcome timing may all influence the observed associations. While a pre-specified outcome hierarchy was applied, this variability should be considered when interpreting the pooled estimates.

Third, considerable clinical heterogeneity existed in terms of surgical populations, ranging from vascular surgery to thoracic, transplant, and general noncardiac procedures. These groups carry substantially different baseline cardiac risks, and the pooled estimates should therefore be interpreted as summary estimates across a broad spectrum rather than predictions applicable to any specific surgical population. This heterogeneity may have materially influenced the magnitude of pooled associations and limits the generalizability of the findings to individual clinical settings. Fourth, although CT-FFR demonstrated a strong association with perioperative MACE, evidence was limited to a single study; this component of the analysis should be considered exploratory and descriptive rather than a robust systematic synthesis, and the title, aims, and conclusions have been revised accordingly. Fifth, the pooling of HRs and ORs, while supported by the low observed event rates (2–9%), represents a methodological limitation; sensitivity analyses stratified by effect measure type were planned but limited by the small number of eligible studies. Sixth, only a subset of identified CCTA studies contributed to the quantitative analysis, as several did not report data in a form amenable to pooling; this selection may have introduced additional bias. Seventh, formal assessment of publication bias using funnel plot asymmetry and Egger’s test was feasible only for the CAC analysis (≥5 studies); for CCTA and CT-FFR analyses, the small number of eligible studies precluded formal testing, and small-study effects cannot be excluded. This represents an important limitation of the quantitative synthesis.

In addition, the handling of elective preoperative revascularisation prompted by CT findings varied inconsistently across included studies, and was not explicitly reported in several cases. Where elective preoperative revascularisation was performed following CT findings and not counted as a MACE event, a potential reduction in observed event rates may have attenuated the magnitude of the prognostic associations. This represents an important source of outcome heterogeneity that could not be fully addressed in the present analysis and is now detailed in Table S1.

Finally, publication bias cannot be entirely excluded, as studies with positive findings may be more likely to be published.

## 5. Conclusions

CT-based coronary imaging markers are significantly associated with perioperative MACE in patients undergoing noncardiac surgery. In particular, obstructive CAD on CCTA and CAC burden demonstrated robust and consistent prognostic associations across multiple studies, while CT-FFR showed a strong exploratory signal in a single study.

These findings are consistent with a pattern of numerically increasing prognostic estimates from plaque burden (CAC) to anatomic stenosis (CCTA), with an exploratory signal from a single CT-FFR study suggesting a potential role for functional ischemia assessment. The concept of a formal hierarchy across modalities requires confirmation in larger prospective studies before definitive conclusions can be drawn. CT-based coronary imaging may therefore provide valuable complementary information for preoperative cardiovascular risk assessment.

Further prospective studies are needed to define the optimal role of CT-based imaging strategies in guiding perioperative management.

## Figures and Tables

**Table 1:** Characteristics of Studies Included in the Systematic Review.

Study (Year)	Country	Study Design	Population/Surgery Type	Sample Size (N)	CT Modality (Scanner)	Primary CT Variables	Follow-Up
Mahla (2001) [8]	Austria	Prospective	Elective vascular surgery	326	EBCT (Imatron C-150)	CACS	Perioperative
Ghadri (2012) [9]	Switzerland	Prospective	Elective noncardiac surgery	326	CACS + SPECT (64-slice CT, GE Healthcare)	CACS	40 days
Ahn (2013) [10]	Korea	Prospective	Intermediate-risk noncardiac	239	CCTA + CACS	CAD severity, CACS	30 days
Chang (2014) [12]	Korea	Prospective	Vascular surgery	192	CCTA (+Stress)	Stenosis severity	30 days
Kong (2015) [11]	USA	Retrospective	Liver transplantation	443	CACS	CACS	30 days
Sheth (2015) [13]	Multinational	Prospective	Major noncardiac surgery	955	CCTA	Stenosis severity	30 days
Hwang (2015) [14]	Korea	Prospective	Noncardiac surgery	484	CCTA	CAD extent/severity	30 days
Shalaeva (2016) [15]	Russia	Prospective	Diabetic/Amputation	119	CCTA + CACS	CAD severity, CACS	Perioperative
PANDA (2020) [16]	Korea	Prospective	Noncardiac surgery	206	CCTA + CACS	Stenosis, CACS	30 days
Yang (2022) [17]	China	Retrospective	Lung cancer surgery	4491	Non-gated CT	CAC burden	30 days
ENCORES (2023) [18]	USA	Retrospective	Major noncardiac surgery	2554	Non-gated CT	Estimated CAC burden	30 days
Ma (2024) [19]	China	Retrospective	Lung cancer surgery	318	CCTA + CT-FFR	CT-FFR	30 days
Park (2026) [20]	Korea	Prospective	Noncardiac surgery	447	CCTA + CACS	Stenosis, CACS	30 days

Abbreviations: CAC, coronary artery calcium; CAD, coronary artery disease; CCTA, coronary computed tomography angiography; CT-FFR, CT-derived fractional flow reserve; SPECT, single-photon emission computed tomography. Scanner details: CT scanner models were not uniformly reported across included studies. Where reported, scanners included electron-beam CT (Imatron C-150), 64-slice CT systems (GE LightSpeed VCT; Siemens Somatom Definition; Philips Brilliance 64), and 128-slice dual-source CT. Studies not reporting scanner specifications are indicated as not reported (NR) in the modality column.

**Table 2:** Risk of Bias Assessment of Included Studies (QUIPS Tool).

Study	Study Participation	Study Attrition	Prognostic Factor Measurement	Outcome Measurement	Confounding Adjustment	Statistical Analysis	Overall Risk
Mahla (2001) [8]	Moderate	Low	Moderate	Low	Moderate	Moderate	Moderate
Ghadri (2012) [9]	Low	Low	Low	Low	Moderate	Low	Low
Ahn (2013) [10]	Low	Low	Low	Low	Low	Low	Low
Chang (2014) [12]	Low	Low	Low	Low	Moderate	Low	Low
Kong (2015) [11]	Moderate	Low	Low	Low	Moderate	Moderate	Moderate
Sheth (2015) [13]	Low	Low	Low	Low	Low	Low	Low
Hwang (2015) [14]	Low	Low	Low	Low	Moderate	Low	Low
Shalaeva (2016) [15]	Moderate	Moderate	Low	Low	Moderate	Moderate	Moderate
PANDA (2020) [16]	Low	Low	Low	Low	Low	Low	Low
Yang (2022) [17]	Low	Low	Moderate	Low	Moderate	Low	Low
ENCORES (2023) [18]	Low	Low	Moderate	Low	Moderate	Low	Low
Ma (2024) [19]	Moderate	Low	Low	Low	Moderate	Low	Low
Park (2026) [20]	Low	Low	Low	Low	Low	Low	Low

Risk of bias was assessed using the Quality In Prognosis Studies (QUIPS) tool. Each domain was classified as low, moderate, or high risk of bias. Abbreviations: QUIPS, Quality In Prognosis Studies.

**Table 3:** Pooled Prognostic Impact of CT-Based Coronary Imaging Markers for Perioperative MACE.

Imaging Modality	Pooled OR	95% CI	Interpretation
Coronary artery calcium (CAC)	2.48	1.76–3.50	Plaque burden
Obstructive CAD on CCTA	7.18	3.89–13.25	Anatomic stenosis
CT-derived fractional flow reserve (CT-FFR)	10.77	4.64–25.02	Functional ischemia

Pooled estimates were derived from random-effects meta-analysis when two or more studies were available. The CT-FFR estimate derives from a single study and is exploratory; direct comparison with pooled CAC and CCTA estimates should be interpreted with caution. Abbreviations: CAC, coronary artery calcium; CCTA, coronary computed tomography angiography; CT-FFR, CT-derived fractional flow reserve; MACE, major adverse cardiac events; OR, odds ratio; CI, confidence interval.

## Data Availability

The data supporting the reported results are available from the corresponding author upon reasonable request.

## Supplementary Materials

The following supporting information can be downloaded at: https://www.mdpi.com/article/10.3390/jcm15166290/s1, Table S1: MACE Definitions and Revascularisation Handling Across Included Studies; Table S2: PRISMA 2020 Checklist.
