# Supplementary material for: Prognostic Value of CT-Based Coronary Imaging for Perioperative Cardiovascular Risk Stratification Before Noncardiac Surgery: An Updated Systematic Review and Meta-Analysis
Source: J Clin Med. 2026 Aug 14;15(16):6290. doi: 10.3390/jcm15166290 (PMC13513533; doi:10.3390/jcm15166290)
Supplement: Supplementary file 1 [file jcm-15-06290-s001.zip › jcm-4459822-supplementary.pdf]

Table S1. MACE Definitions and Revascularisation Handling Across Included Studies

Study (Year)	MACE Definition	Outcome Timing	Revascularisation Handling
**Mahla (2001)**	Cardiac death, nonfatal MI, unstable angina requiring hospitalisation	Perioperative (in-hospital)	Not included in MACE; elective PCI/CABG prior to surgery led to exclusion or separate reporting
**Ghadri (2012)**	Cardiac death, nonfatal MI, unstable angina, heart failure requiring hospitalisation	40 days	Urgent revascularisation included; elective preoperative revascularisation not reported separately
**Ahn (2013)**	Cardiac death, nonfatal MI, unstable angina, heart failure	30 days	Not explicitly reported; preoperative revascularisation not counted as MACE
**Chang (2014)**	Cardiac death, nonfatal MI, unstable angina	30 days	Not included; patients undergoing elective revascularisation prior to surgery were managed per clinical decision without being counted as events
**Kong (2015)**	Cardiac death, nonfatal MI, unstable angina requiring hospitalisation	30 days	Not reported; pre-transplant revascularisation excluded from outcome analysis
**Sheth (2015)**	Cardiac death, MI (troponin-based), unstable angina, acute heart failure	30 days	Urgent revascularisation included as MACE component; elective preoperative revascularisation not explicitly addressed
**Hwang (2015)**	Cardiac death, nonfatal MI, unstable angina, heart failure	30 days	Not included; preoperative management (including

			revascularisation) was at physician discretion and not counted as outcome
**Shalaeva (2016)**	Cardiac death, nonfatal MI, acute heart failure, life-threatening arrhythmia	Perioperative (in-hospital)	Not reported; study population (diabetic amputation) had low revascularisation rate
**PANDA (2020)**	Cardiac death, MI, unstable angina, heart failure hospitalisation, stroke	30 days	Urgent revascularisation included in expanded MACE; elective preoperative revascularisation excluded from outcome count
**Yang (2022)**	Cardiac death, nonfatal MI (MINS included), unstable angina	30 days	Not included; no patients underwent elective preoperative revascularisation based on CT findings
**ENCORES (2023)**	Cardiac death, nonfatal MI, unstable angina, acute HF, cardiac arrest	30 days	Not included; preoperative revascularisation based on CT findings was not performed in this population
**Ma (2024)**	Cardiac death, MI (MINS included), unstable angina requiring hospitalisation	30 days	Not included; CT-FFR-positive patients were referred for further evaluation but revascularisation not counted as MACE
**Park (2026)**	Cardiac death, nonfatal MI, unstable angina, heart failure hospitalisation	30 days	Urgent revascularisation included; elective preoperative revascularisation excluded from primary outcome

Abbreviations: CAC, coronary artery calcium; CABG, coronary artery bypass grafting; HF, heart failure; MACE, major adverse cardiac events; MI, myocardial infarction; MINS, myocardial injury after noncardiac surgery; PCI, percutaneous coronary intervention. NR, not reported.

Table S2: PRISMA 2020 Checklist

Prognostic Value of CT-Based Coronary Imaging for Perioperative Cardiovascular Risk Stratification Before Noncardiac Surgery: An Updated Systematic Review and Meta-Analysis Including CT-Derived Fractional Flow Reserve

Item #	Topic	Checklist Item	Page #
**TITLE**			
1	Title	Identify the report as a systematic review.	1
**ABSTRACT**			
2	Abstract	See the PRISMA 2020 for Abstracts checklist.	1–2
**INTRODUCTION**			
3	Rationale	Describe the rationale for the review in the context of existing knowledge.	3–4
4	Objectives	Provide an explicit statement of the objective(s) or question(s) the review addresses.	4
**METHODS**			
5	Eligibility criteria	Specify the inclusion and exclusion criteria for the review and how studies were grouped for the syntheses.	4–5
6	Information sources	Specify all databases, registers, websites, organisations, reference lists and other sources searched or consulted to identify studies. Specify the date when each source was last searched or consulted.	5
7	Search strategy	Present the full search strategies for all databases, registers and websites, including any filters and limits used.	5
8	Selection process	Specify the methods used to decide whether a study met the inclusion criteria of the review, including how many reviewers screened each record and each report retrieved, whether they worked independently, and if applicable, details of automation tools used in the process.	5
9	Data collection process	Specify the methods used to collect data from reports, including how many reviewers collected data from each report, whether they worked independently, any processes for obtaining or confirming data from study investigators, and if applicable, details of automation tools used in the process.	5
10a	Data items	List and define all outcomes for which data were sought. Specify whether all results that were compatible with each outcome domain in each study were sought (e.g. for all measures, time points, and analyses), and if not, the methods used to decide which results to collect.	5
10b	Data items	List and define all other variables for which data were sought (e.g. participant and intervention characteristics, funding sources).	5

Item #	Topic	Checklist Item	Page #
		Describe any assumptions made about any missing or unclear information.	
11	Study risk of bias assessment	Specify the methods used to assess risk of bias in the included studies, including details of the tool(s) used, how many reviewers assessed each study and whether they worked independently, and if applicable, details of automation tools used in the process.	5–6
12	Effect measures	Specify for each outcome the effect measure(s) (e.g. risk ratio, mean difference) used in the synthesis or presentation of results.	6
13a	Synthesis methods	Describe the processes used to decide which studies were eligible for each synthesis (e.g. tabulating the study intervention characteristics and comparing against the planned groups for each synthesis).	6
13b	Synthesis methods	Describe any methods required to prepare the data for presentation or synthesis, such as handling of missing summary statistics, or data conversions.	6
13c	Synthesis methods	Describe any methods used to tabulate or visually display results of individual studies and syntheses.	6
13d	Synthesis methods	Describe any methods used to synthesise results and provide a rationale for the choice(s). If meta-analysis was performed, describe the model(s), method(s) to identify the presence and extent of statistical heterogeneity, and software package(s) used.	6
13e	Synthesis methods	Describe any methods used to explore possible causes of heterogeneity among study results (e.g. subgroup analysis, meta-regression).	6
13f	Synthesis methods	Describe any sensitivity analyses conducted to assess robustness of the synthesised results.	6
14	Reporting bias assessment	Describe any methods used to assess risk of bias due to missing results in a synthesis (arising from reporting biases).	6
15	Certainty assessment	Describe any methods used to assess certainty (or confidence) in the body of evidence for an outcome.	NA
16	Registration and protocol	Provide registration information for the review, including register name and registration number, or state that the review was not registered. Indicate where the review protocol can be accessed, or state that a protocol was not prepared.	5
**RESULTS**			
17	Study selection	Describe the results of the search and selection process, from the number of records identified in the search to the number of studies included in the review, ideally using a flow diagram.	7, Fig. 1
18	Study characteristics	Cite each included study and present its characteristics.	7–8, Table 1

Item #	Topic	Checklist Item	Page #
19	Risk of bias in studies	Present assessments of risk of bias for each included study.	8, Table 2
20a	Results of individual studies	For all outcomes, present, for each study: (a) summary statistics for each group (where appropriate) and (b) an effect estimate and its precision (e.g. confidence/credible interval), ideally using structured tables or plots.	8–10, Figs. 2–4
20b	Results of individual studies	Report results for all outcomes (benefits and harms) for each intervention (or exposure) being evaluated.	8–10
21a	Results of syntheses	For each synthesis, briefly summarise the characteristics and risk of bias among contributing studies.	8–10
21b	Results of syntheses	Present results of all statistical syntheses conducted. If meta-analysis was done, present for each the summary estimate and its precision, the amount of statistical heterogeneity, and the I^2 statistic.	8–10, Table 3
21c	Results of syntheses	Present results of any investigations of possible causes of heterogeneity among study results.	8–10
21d	Results of syntheses	Present results of all sensitivity analyses conducted to assess the robustness of the synthesised results.	11
22	Reporting biases	Present assessments of risk of bias due to missing results (arising from reporting biases) for each synthesis assessed.	11, 14
23	Certainty of evidence	Present assessments of certainty (or confidence) in the body of evidence for each outcome assessed.	NA
24	Excluded studies	Cite studies that might appear to meet the inclusion criteria, but which were excluded, and explain why they were excluded.	7
**DISCUSSION**			
25	Discussion	Provide a general interpretation of the results in the context of other evidence.	11–13
26	Limitations	Discuss limitations of the review process and results.	13–14
27	Conclusions	Provide a general interpretation of the results in the context of other evidence.	14
**OTHER INFORMATION**			
28	Funding	Describe sources of financial or non-financial support for the review, and the role of the funders or sponsors in the review.	15
29	Competing interests	Declare any competing interests of review authors.	15
30	Availability of data	Report which of the following are publicly available and where they can be found: template data collection forms; data extracted from included studies; data used for all analyses; analytic code; any other materials used in the review.	15

From: Page MJ, McKenzie JE, Bossuyt PM, Boutron I, Hoffmann TC, Mulrow CD, et al. The PRISMA 2020 statement: an updated guideline for reporting systematic reviews. BMJ 2021;372:n71. doi: 10.1136/bmj.n71. NA = Not applicable.
